# Serum albumin, a good indicator of persistent organ failure in acute pancreatitis

**DOI:** 10.1186/s12876-017-0615-8

**Published:** 2017-04-26

**Authors:** Shoukang Li, Yushun Zhang, Mengjiao Li, Chao Xie, Heshui Wu

**Affiliations:** 1 Pancreatic Disease Institute, Union Hospital, Tongji Medical College, Huazhong University of Science and Technology, 430022 Wuhan, Hubei Province People’s Republic of China; 2 Department of Endocrinology, Union Hospital, Tongji Medical College, Huazhong University of Science and Technology, 430022 Wuhan, Hubei Province People’s Republic of China

**Keywords:** Acute pancreatitis, Persistent organ failure, Albumin

## Abstract

**Background:**

To evaluate the predictive value of serum albumin (ALB) for persistent organ failure (POF) in acute pancreatitis (AP).

**Methods:**

We selected 158 patients with AP in this retrospective study from Jan.1st, 2015 to Dec.31st, 2015. Forty-six patients were diagnosed with POF. All the values of laboratory parameters were measured upon admission to hospital. And 48 h after admission, we examined serum albumin of each patient again, called ‘ALB2’. Uni-and multi-variate logistic regression were used to evaluate the impact of ALB to predict POF.

**Results:**

The median age of the whole population was 48 years and 53.8% were male. The admission-time albumin of AP patients with POF was distinctly lower than patients without POF (28.9 (25.3–33.1) g/L vs. 38.5 (34.0–40.1) g/L, *p* < 0.001). In uni-variate analysis, WBC, PT, GLU, LDH, ALB, ALB2, BUN, Ca, HDL-C and Ranson were significantly associated with POF. After multivariate regression, ALB remained an independent prognostic factor for POF in AP (OR: 0.748, 95%CI: 0.645–0.868; *p* < 0.05). The AUC for ALB is 0.873 (0.808, 0.938), even larger than that for Ranson, 0.845 (0.634, 0.913).

**Conclusions:**

We identified serum albumin predictive to persistent organ failure in acute pancreatitis.

## Background

Acute pancreatitis (AP) is known to be an inflammatory disorder which has a process observed clinically from local pancreatitis through the systemic inflammatory response, organ dysfunction and death. Minority patients will develop a severe disease with local or systemic complications even organ failure (OF) while most patients suffer from a mild, self-limiting inflammatory process [1]. The severe AP (SAP) has been redefined as AP with persistent organ failure (OF lasts more than 48 h) with a lethality rate of 20–50% according to 2012 revised Atlanta classification for AP [2–7].

It is vital for the determination of therapeutic strategy to have an early assessment of disease severity because effective treatment could significantly decrease mortality of patients with severe pancreatitis [8, 9]. Some invasive or non-invasive methods, including scoring systems, radiological imaging modalities, and biochemical parameters are used for diagnosing and evaluating disease severity of AP.

Abnormal low-level of albumin signals act as a pivotal starter in the pathogenesis of AP. The hypoproteinemia has been observed in AP patients and the mechanism was studied too. There are also some therapy aimed at albumin having been used on AP patients [10]. However, with the publication of revised Atlanta classification for AP, the relationship between incidence of POF in the AP pathophysiology and serum albumin has not been assessed yet. Our study was aimed to evaluate the value of serum albumin on admission of hospital in correlation with the incidence of POF in AP.

## Methods

### Patients

In total, 158 patients with AP were recruited in our study from Jan.1st, 2015 to Dec.31st, 2015, in the Pancreatic Disease Institute of Wuhan Union Hospital. Diagnosis of AP was based on clinical findings based on the presence of two or more of the following three criteria: 1) abdominal pain consistent with AP; 2) contrast-enhanced computed tomography (CECT), magnetic resonance imaging (MRI) or abdominal ultrasonography findings characteristic of AP; 3) serum amylase and/or lipase elevation ≥ three times the upper limit of normal [2]. The exclusion criteria included any of the following: 1) Patients younger than 18-year-old; 2) the time from abdominal pain onset to hospital admission ≥ 72 h; 3) chronic pancreatitis; 4) pancreatitis induced by trauma or pregnancy; and 5) unavailable laboratory measurements or medical records. Laboratory data were obtained from the blood screening test at hospitalization. Patient’s paper charts and electronic medical records were reviewed for information on demographics, physiologic variable, and disease severity by one independent physician. The study was conducted according to the principles of the Declaration of Helsinki. For the reason that all data were retrieved retrospectively from the laboratory test information system without additional laboratory analysis or blood samples, so that informed consent for individual patient was not obtained. This study was approved by the ethics review board of Wuhan Union Hospital.

### Definition

OF was diagnosed according to Modified Marshall score as described in the revised 2012 Atlanta classification when the following cutoffs were exceeded: 1) respiratory failure if the ratio of PaO2/FiO2 was < 300 mmHg; 2) renal failure if serum creatinine was ≥ 1.9 mg/dl; and 3) cardiovascular failure if systolic blood pressure was < 90 mmHg despite fluid replacement. POF was identified when organ failure lasts for more than 48 h [2].

### Statistic analysis

Statistic analysis has been done by SPSS 17.0 (SPSS Inc, Chicago IL, USA). Data were tested for normality and were found to be nonnormally distributed. Accordingly, all the data are presented as median value with interquartile range. All the collected factors were analyzed by ‘Mann-Whitney U’ test or *χ*2 test first. And then, the remaining valuable factors were chosen out and joined in Binary Logistic Regression. We used both ‘univariate analysis’ and ‘multivariate analysis’ to test 10 remaining factors together with age. We also used ROC line to describe the value in prediction of albumin. A *P* value < 0.05 was considered to statistically significant.

## Results

### Patient characteristics

All 158 patients were collected from Jan.1st, 2015 to Dec.31st, 2015 in the Pancreatic Disease Institute of Wuhan Union Hospital. The male-female ratio (85/73) was 1.16, with a median age of 48 years. 46 patients had POF at last.

There were 30 patients developing solitary POF (all of respiratory system) while the other 16 patients developing multiple POF (11 of lung and kidney, 3 of lung and heart, and 2 of lung, kidney and heart). 15 patients with POF died with an overall mortality of 9.5% during hospitalization. No death was observed in patients without POF (Table 1).

**Table 1:** Types of persistent organ failure and the corresponding mortality

	Persistent organ failure	In-hospital mortality
Solitary POF	30	6
Respiratory	30	6
Renal	0	0
Cardiovascular	0	0
Multiple POF	16	9
Respiratory + renal	11	5
Respiratory + cardiovascular	3	2
Respiratory + cardiovascular + renal	2	2
Total	46	15

### Comparison between patients with and without POF

Compared to patients without POF, patients with POF showed significantly elevated values of WBC, PT, GLU, LDH, BUN, and Ranson score, whereas the levels of ALB, ALB2 (serum albumin after 48 h after admission), Ca, HDL-C were statistically lower (Table 2).

**Table 2:** Result of mean analysis for univariate

	Without POF (POF = 0),*n* = 112	With POF (POF = 1),*n* = 46	*P*-value
Sex (M/F)	60/52	25/21	NS
Age (year)	48 (39–58)	49 (38–63)	NS
WBC (G/L)	12.05 (9.56–15.14)	13.91 (11.30–18.09)	0.001
MPV (fl)	10.0 (9.3–11.1)	10.5 (9.5–11.6)	NS
PLT (G/L)	205 (153–239)	163 (113–229)	NS
PT (s)	13.5 (12.8–14.6)	15.4 (14.7–16.3)	0.000
GLU (mmol/L)	6.8 (5.8–9.1)	10.0 (7.8–14.1)	0.000
LDH (U/L)	251 (184–360)	470 (363–669)	0.000
Tbil (umol/L)	23.6 (14.6–35.7)	31.1 (16.5–43.7)	NS
ALT (U/L)	38.5 (25.0–117.8)	40.0 (25.8–79.0)	NS
AST (U/L)	36.0 (21.0–169.3)	44.5 (30.0–116.8)	NS
ALP (U/L)	76.0 (62.3–121.8)	72.0 (56.8–103.0)	NS
GGT (U/L)	80.0 (34.3–288.5)	78.0 (34.5–164.3)	NS
ALB (g/L)	38.5 (34.0–40.1)	28.9 (25.3–33.1)	0.000
ALB2 (g/L)	33.6 (31.0–38.5)	26.0 (22.5–28.5)	0.000
BUN (mmol/L)	4.65 (3.30–5.50)	7.30 (4.65–10.71)	0.000
Ca (mmol/L)	2.15 (2.02–2.26)	1.76 (1.41–1.93)	0.000
HDL-C (mmol/L)	1.01 (0.75–1.26)	0.47 (0.34–0.92)	0.000
Ranson	3 (1–4)	5 (4–6)	0.000

Data are presented as median value (interquartile range)

*ALB* albumin, *ALB2* serum albumin after 48 h after admission, *ALP* alkaline phosphatase, *ALT* alanine aminotransferase, *AST* aspartate amino transferase, *BUN* Blood urea nitrogen, *Ca* calcium, *GGT* γ-glutamyl transpeptidase, *GLU* fasting blood-glucose, *HDL-C* high density lipoprotein cholesterol, *LDH* lactate dehydrogenase, *MPV* mean platelet volume, *PLT* platelets, *PT* Prothrombin Time, *Ranson* Ranson-score, *Tbil* total bilirubin, *WBC* white blood cell, *NS* not significant

### Serum albumin as an independent indicator of persistent organ failure in acute pancreatitis

We put those 10 meaningful factors together with age into univariate logistics regression model. In univariate analysis, all 10 factors were found to be significantly associated with the incidence of POF. For the fact that WBC, GLU, LDH, Ca, BUN are included in Ranson-score system, ALB and ALB2 are closely related, Multivariate Analysis was then performed using PT, ALB, HDL-C and Ranson. According to the results, ALB remained an independent prognostic factor for POF in AP (OR: 0.748, 95%CI: 0.645–0.868; *p* < 0.05) (Table 3).

**Table 3:** Uni- and multivariate logistics regression analysis of risk factors to POF

	Univariate analysis		Multivariate analysis	
	Odds ratio (95%CI)	*P*-value	Odds ratio (95%CI)	*P*-value
Age	1.012 (0.988, 1.037)	NS	-	
WBC	1.128 (1.043, 1.219)	0.003	Parameter of Ranson-score system	
PT	2.002 (1.518, 2.640)	0.000	1.182 (0.784, 1.783)	NS
GLU	1.247 (1.125, 1.382)	0.000	Parameter of Ranson-score system	
LDH	1.008 (1.005, 1.011)	0.000	Parameter of Ranson-score system	
ALB	0.731 (0.661, 0.808)	0.000	0.748 (0.645, 0.868)	0.000
ALB2	0.642 (0.555, 0.743)	0.000	-	
BUN	1.345 (1.171, 1.544)	0.000	Parameter of Ranson-score system	
Ca	0.003 (0.000, 0.021)	0.000	Parameter of Ranson-score system	
HDL-C	0.070 (0.022, 0.217)	0.000	0.384 (0.074, 1.984)	NS
Ranson	2.529 (1.838, 3.479)	0.000	2.219 (1.410, 3.493)	0.001

WBC, GLU, LDH, BUN, Ca are parameter of Ranson-score system, we choose Ranson rather than WBC, GLU, LDH, BUN, Ca. ALB and ALB2 are closely related, we only choose ALB

**Fig. 1:**
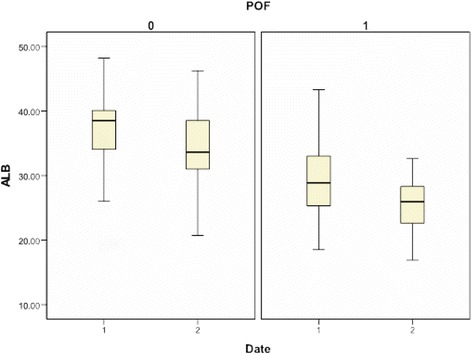
Difference of ALB and ALB2 between patients with and without POF POF: ‘0’ = without POF, ‘1’ = with POF; Date: ‘1’ = at admission, ‘2’ = 48 h after admission

A boxplot was drawn to show the difference of ALB and ALB2 between patients with and without POF (Fig. 1).

### Predictive value of serum albumin for POF

**Fig. 2:**
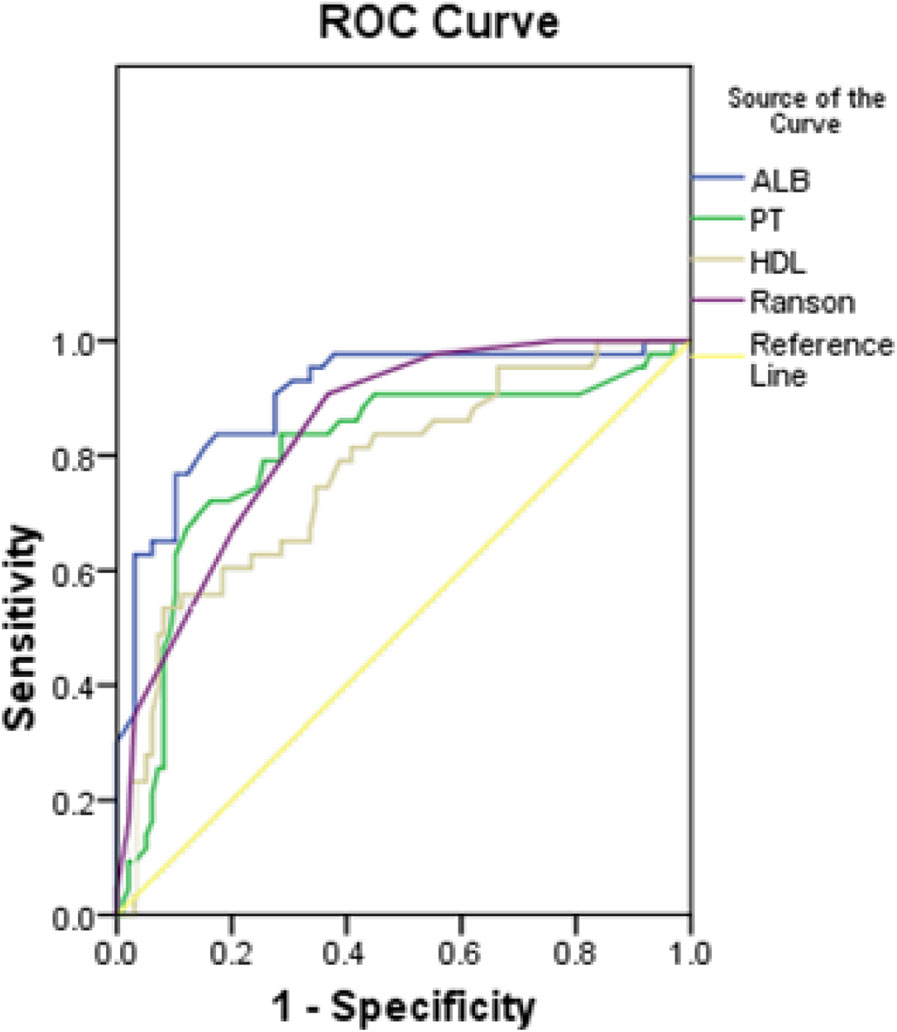
ROC-line of PT, ALB, HDL-C and Ranson-score

The ‘ROC line’ of PT, ALB, HDL-C and Ranson-score were shown (Fig. 2).

As demonstrated (Table 4), ALB on admission was shown to have an area under curve (AUC) of the receiver operating characteristic of 0.873 (95%CI: 0.808–0.938), with specificity of 83.0%, a sensitivity of 78.3%, positive predictive value (PPV) of 65.5%, and negative Predictive value (NPV) of 90.3%. The optimal threshold was 33.3 g/L. The AUC of PT, HDL-C and Ranson score were 0.800 (95%CI: 0.715, 0.884), 0.769 (0.684, 0.854), 0.845 (95%CI: 0.634–0.913) respectively. It proved that albumin is a better predictor compared to other simpler predictors and severity scores in predicting POF in AP.

**Table 4:** AUC for ALB and Ranson-score

	AUC (95%CI)	Cut-off	sensitivity	specificity	PPV	NPV
PT	0.800 (0.715, 0.884)	15.0	0.717	0.848	0.660	0.879
ALB	0.873 (0.808, 0.938)	33.3	0.783	0.830	0.655	0.903
HDL-C	0.769 (0.684, 0.854)	0.50	0.535	0.918	0.741	0.818
Ranson	0.845 (0.634, 0.913)	4.00	0.913	0.634	0.506	0.947

*AUC* Area Under The Curve, *NPV* negative Predictive value, *PPV* positive predictive value

## Discussion

Acute pancreatitis is one of the most serious emergencies in abdominal surgery department. Although we have met amount of patients with acute pancreatitis who got satisfactory results, we still have to pay attention to this disease for its high mortality especially in severe type. We designed our research according to 2012 revision of the Atlanta Classification of acute pancreatitis, and we found the level of albumin in acute pancreatitis patients with POF is much lower than patients without POF.

Albumin, with 585 residues, and three domains of similar size, each one containing two sub-domains, is a stable and very flexible heart-shape-molecule [11]. It’s a natural plasma protein of which the median half-life is around 18 to 19 days and synthesized only by liver at a rate of 9 to 14 g per day in healthy individuals [12]. However, albumin can be catabolized in most organs of the body at a similar rate by uptake into endocytotic vesicles on the endothelial surface and finally turn into amino acids as breakdown products [12, 13].

Albumin has well-known effects on maintaining fluid balance, being responsible for 75 to 80% of colloid osmotic pressure in the basal physiological state [12, 14]. Albumin can bind to an extremely wide range of endogenous and exogenous ligands, to transport them to specific tissues and organs, to increase their solubility in plasma, or to dispose of them when they are toxic. The chemical structure of albumin can be altered by some specific processes (oxidation, glycation) leading to rapid clearance and catabolism [11].

In the progress of many diseases, including cancer, infection, inflammation, a low-level of serum albumin has been identified [15–18]. According to the researches into the phenomenon of low-level of serum albumin in those diseases, abnormal metabolism of albumin led by inflammation response may be an important reason for that, as well as a low intake of albumin [19]. The mechanism is not clear yet, however, IL-6 and other inflammatory cytokines may play an important role in it [11, 18]. Similarly, this phenomenon has also been found in AP, especially obvious in severe acute pancreatitis patients. The mechanism is complicated and not so clear yet. But some researches also gave explanation, that (a) The reaction in the progress of SAP, including infection, will lead to insulin resistance, which may at last result in metabolic disorders. Thus, the degradation of albumin gets much more for the reason of lower using-rate of glucose and fat; (b) The ability of biosynthesis of albumin in liver is weak for low intake and stimulate of inflammatory factors; (c) In the progress of stress response, vasopermeability become higher so that albumin will permeate into tissue space [11, 13, 15, 20, 21].

POF, which develops in 10–20% of AP patients with a mortality rate between 20 and 50%, is the most important cause of death within the first 2 weeks of disease onset [2, 3]. It’s critical to have the ability to assess the risk of AP patients developing POF earlier upon hospitalization, both for triaging patients to the appropriate grade of care and for designing appropriate intervention and medical treatment [22]. Lots of invasive or non-invasive methods, including biochemical parameters, severity scores and radiological imaging modalities have been applied for predicting POF in patients with AP. In a systemic review for prospective studies, the Bedside Index of Severity in Acute Pancreatitis (BISAP) and the Japanese Severity Score were identified as the best predictors evaluating predictors of POF within 48 h of admission [23]. The Japanese Severity Scale showed a specificity of 0.90 with a cutoff value of 1. But they were too cumbersome to be applied in the individual patient. The Japanese Severity Scale was made up of 21 parameters, and the BISAP included a total of 8 parameters. This highlights the need to develop approaches to prediction of POF that have early practical utility while still providing a performance sufficient to be applied in the individual patient. Another study found that the Glasgow score is the best classifier at 48 h of admission for predicting POF in patients with AP by using a head-to-head comparison between the Ranson, APACHE II, Glasgow and the BISAP scoring systems [24]. Mounzer et al. compared several existing clinical scoring systems and found that these scores showed modest accuracy (AUC at admission of 0.6 to 0.8 in both the training and the validation cohorts) and seemed to have reached their maximal efficacy to predict POF in patients with AP [25].

In our research, albumin always descends obviously in AP patients with POF (*p* < 0.05). The AUC under ROC line is 0.873. Albumin has been proved as an excellent marker of POF in AP. However, no previous study has researched into the association between albumin and incidence of POF in AP. Therefore, this study is the first time to show that the reduction of serum albumin is significantly associated with increased risk of POF in AP.

Though the boxplot showed that low-level of ALB2 also had a closely relation to the development of POF on AP patients, the prediction function of ALB2 is not reliable: (1). Patient with low-level serum albumin 48 h after admission may have been in POF state, that is low-level ALB 48 h later could be the result of POF rather than an indicator. (2). During the 48 h in hospital, different therapies were used including infusion of human serum albumin, which can lead to error in our research. (3). The indicator of POF need to help us make decision as soon as we checked our patients, 48-h is quite a long time.

However, the intra-individual variation in albumin value can be another interesting study to be addressed.

There are several limitations of the present study. The sample size of our study is a bit small. And the causality role of POF and albumin in AP, however, requires to be investigated further in a prospective validation study as it is an observational study.

## Conclusions

In conclusion, our present study reveals that serum albumin on admission is independently associated with POF in AP. We suggest that albumin is a valuable tool for a rapid assessment of POF in patients with AP.

## Abbreviations

ALB: Albumin at admission
ALB2: Serum albumin after 48 h after admission
ALP: Alkaline phosphatase
ALT: Alanine aminotransferase
AP: Acute pancreatitis
AST: Aspartate amino transferase
AUC: Area under the curve
BUN: Blood urea nitrogen
Ca: Calcium
GGT: γ-glutamyl transpeptidase
GLU: Fasting blood-glucose
HDL-C: High density lipoprotein cholesterol
LDH: Lactate dehydrogenase
MPV: Mean platelet volume
NPV: Negative predictive value
NS: Not significant
PLT: Platelets
POF: Persistent organ failure
PPV: Positive predictive value.
PT: Prothrombin time
Ranson: Ranson-score
SAP: Severe acute pancreatitis
Tbil: Total bilirubin
WBC: White blood cell
