# Erratum to: Serum albumin, a good indicator of persistent organ failure in acute pancreatitis

**DOI:** 10.1186/s12876-017-0645-2

**Published:** 2017-07-07

**Authors:** Shoukang Li, Yushun Zhang, Mengjiao Li, Chao Xie, Heshui Wu

**Affiliations:** 1 Department of Pancreatic Surgery, Union Hospital, Tongji Medical College, Huazhong University of Science and Technology, Wuhan, 430022 China; 2 Department of Endocrinology, Union Hospital, Tongji Medical College, Huazhong University of Science and Technology, Wuhan, 430022 China

## Erratum

Following publication of this article [1], it has come to our attention that the affiliations were captured as:

1 Pancreatic Disease Institute, Union Hospital, Tongji Medical College, Huazhong University of Science and Technology, 430022 Wuhan, Hubei Province, People’s Republic of China. 2 Department of Endocrinology, Union Hospital, Tongji Medical College, Huazhong University of Science and Technology, 430022 Wuhan, Hubei Province, People’s Republic of China.

However, the correct affiliations should be as following:

1 Department of Pancreatic Surgery, Union Hospital, Tongji Medical College, Huazhong University of Science and Technology, Wuhan 430022, China.

2 Department of Endocrinology, Union Hospital, Tongji Medical College, Huazhong University of Science and Technology, Wuhan 430022, China.
